# Ureteral Stent Placement Increases the Risk for Developing BK Viremia after Kidney Transplantation

**DOI:** 10.1155/2014/459747

**Published:** 2014-09-11

**Authors:** Faris Hashim, Shehzad Rehman, Jon A. Gregg, Vikas R. Dharnidharka

**Affiliations:** ^1^Divisions of Pediatric Nephrology, University of Florida College of Medicine, Gainesville, FL, USA; ^2^Transplant Nephrology, University of Florida College of Medicine, Gainesville, FL, USA; ^3^St. Louis Children's Hospital-Division of Pediatric Nephrology, Washington University School of Medicine, St. Louis, MO 63110-1093, USA

## Abstract

The placement of ureteral stent (UrSt) at kidney transplantation reduces major urological complications but increases the risk for developing nephropathy from the BK virus. It is unclear whether UrSt placement increases nephropathy risk by increasing risk of precursor viral replication or by other mechanisms. We retrospectively investigated whether UrSt placement increased the risk for developing BK Viremia (BKVM) in adult and pediatric kidney transplants performed at the University of Florida between July 1, 2007, and December 31, 2010. In this period all recipients underwent prospective BKV PCR monitoring and were maintained on similar immunosuppression. Stent placement or not was based on surgeon preference. In 621 transplants, UrSt were placed in 295 (47.5%). BKVM was seen in 22% versus 16% without UrSt (*P* = 0.05). In multivariate analyses, adjusting for multiple transplant covariates, only UrSt placement remained significantly associated with BKVM (*P* = 0.04). UrSt placement significantly increased the risk for BKVM. Routine UrSt placement needs to be revaluated, since benefits may be negated by the need for more BK PCR testing and potential for graft survival-affecting nephritis.

## 1. Introduction

BK virus (BKV) was first isolated in 1971 from the urine of a Sudanese renal transplant recipient who presented with ureteral stenosis [1]. Years later, a new era in the study of BKV began when BK nephropathy (BKN) was diagnosed by a needle biopsy in a renal transplant (RTx) recipient suspected of having acute rejection [2]. In the following years, additional cases were reported from kidney transplant centers worldwide [3–5]. In nonimmunosuppressed hosts, BKV infection remains latent and asymptomatic in uroepithelial cells, though a fraction of asymptomatic seropositive subjects shed virus into the urine [6]. In states of immunosuppression, such as after kidney transplantation, BKV is reactivated and infection can progress from viruria to viremia, followed by nephropathy [7]. Efforts to eradicate this problem have included the identification of risk factors, early detection of BK viruria (BKVU) and BK viremia (BKVM) through serial PCR screening, early diagnostic biopsy for allograft dysfunction, minimization of immunosuppression for biopsy-proven BKN, and the employment of pharmacotherapy [8].

Risk factors for BKV infection include a higher degree of human leukocyte antigen mismatch, pediatric status, aggressive immunosuppressive regimen, and transplant ureteral stent use [9]. The role of stents is controversial. In two prior single center adult studies, the placement of ureteral stent (UrSt) at the time of kidney transplant was associated with 4-fold increase in the risk for developing BKVN [10, 11]. Our previous pediatric study also showed a 4-fold higher risk, but this result did not reach statistical significance due to limited single center sample size [12]. In this era, the precursor viral replication stages BK viremia (BKVM) and BK viruria (BKVU) were not monitored. It was unclear from these studies whether the increased BK virus nephropathy (BKVN) risk with UrSt placement was secondary to increased likelihood of viral replication or due to other factors unrelated to, or after, replication initiation. Two studies reported an increased risk in the precursor viral replication stage BKVM with UrSt placement at kidney transplant. In both studies, BKVU was not assessed [13, 14]. A twelve-month prospective multicenter study that randomized renal transplant patients to cyclosporine or tacrolimus showed that BKV viremia increased in recipients with any of: higher corticosteroids, using tacrolimus compared to cyclosporine, older age and male gender at month 12 post-transplant [15].

Diagnosing a direct effect of UrSt placement is difficult since by the time there is a clinical correlation such as acute renal failure, hydronephrosis, and ureteral stenosis there is so much fibrosis that BK is not evident. Animal studies have confirmed this [16].

After our adult and pediatric kidney transplant programs instituted a universal BKV serum screening protocol, our practice has been to reduce immunosuppression at time of BK viremia. This has led to a change in the natural history of BKV infection, with nephropathy much less frequent but viruria and viremia commonly detected.

The purpose of this study was to determine the association between transplant UrSt placement and both BKVM/BKVU in a larger group of recipients at a center that performs a large number of adult and pediatric kidney transplants. We report the largest series to date demonstrating the relationship between transplant ureteral stenting and early stages of BKV infection.

## 2. Methods

After approval from the Shands Hospital at University of Florida Institutional Review Board, we conducted a retrospective secondary data analysis of all eligible adult and pediatric renal transplants conducted at our institution between July 1, 2007, and December 31, 2010. All eligible subjects had to have at least 15 days of graft survival and at least 12 months of follow up; otherwise those cases were censored out. At our center, all ureteroneocystostomies during the study time period were performed by extravesical (Lich-Gregoir) technique. All indwelling stents placed were 6-French 12 cm double-J type. All transplants were performed by four surgeons in which certain surgeons almost always stented, whereas others never stented, and that choice of surgeon was arbitrary. Transplant ureteral stents were removed 6–8 weeks following transplantation. We extracted data on recipient demographics (age, sex, race, and primary diagnosis), donor demographics (age, sex, and race), donor source (living or deceased), transplant characteristics (ureteral stent or not, PRA, HLA mismatch, ischemia times, and DGF or not), and initial posttransplant immunosuppression.

During this period, immunosuppression medication consisted of induction with basiliximab (20 mg on days 0 and 3) or rabbit anti-thymocyte globulin (Thymoglobulin, Genzyme, Cambridge, Massachusetts, USA; 3 doses of 1.5 mg/kg/dose for high risk patients). Maintenance medication included prednisone 1 mg/kg/day, postoperatively, which will be tapered slowly over time (steroids beyond the first week were reserved for specific situations in pediatric recipients), calcineurin inhibitor (tacrolimus or cyclosporine) with the dosing adjusted according to the 12-hour trough level, and mycophenolate mofetil (MMF) 2 g/day for Caucasians or 3 g/day for African-Americans (AA) recipients. Pediatric doses for MMF were 600 mg/m^2^ divided bid standard pediatric dose for non-AA children or 750 mg/m^2^ divided bid for AA children. In case mycophenolate sodium was used, the dose was 1440 mg/day divided bid (400 mg/m^2^ for pediatric dose). Both mycophenolate mofetil and mycophenolate sodium are lumped and abbreviated as MMF in this report. Delayed graft function was defined as hemodialysis within 1 week after kidney transplant.

Acute rejection was defined as biopsy-proven rejection. Acute cellular rejection was treated with intravenous (IV) methylprednisolone or rabbit anti-thymocyte globulin (ATG) depending on the severity. On the other hand, antibody mediated rejection was treated with plasmapheresis ± IV methylprednisolone.

Surgical complications assessed included urinary leakage defined as accumulation of urine around the graft and required Foleys catheter insertion with percutaneous nephrostomy, urethral obstruction defined as impairment of graft function with hydronephrosis, or pelvicaliceal dilatation requiring percutaneous nephrostomy insertion or ureteral operation.

### 2.1. Polyomavirus BK Viremia and Viruria Screening Protocol

Beginning one month after transplantation, all renal transplant recipients underwent monthly serum BK polymerase chain reaction (PCR) measurement. Screening for BKV in blood and urine was done monthly for the first year. All PCR assays were performed at Shands Hospital Clinical Laboratories. According to our lab, ≥500 copies/mL indicates viremia and ≥4000 indicates viruria. Significant viremia, at which point we reduced immunosuppression, was generally at ≥5000 copies/mL through the time period of this study. Confirmation of BKVM was defined by detection of BKV PCR in plasma on 2 consecutive positive plasma samples within 2 weeks. The diagnosis of nephropathy (BKVN) was made by typical histological demonstration of BKV within tissue (nuclear positivity of epithelial cells by BKV-specific in situ hybridization or immunostaining for BKV using a mouse anti-BKV large T antigen monoclonal antibody) and organ damage (interstitial infiltrate or elevated serum creatinine).

In many patients where BKVM was confirmed, reduction of immunosuppression was usually initiated by 50% dose reduction of MMF and prednisone; persistent BKVM without impairment of graft function prompted discontinuation of MMF ± tapering of the dose of calcineurin inhibitor. Renal biopsy was performed in any patient with impairment of graft function with starting anti-BK therapy including leflunomide or cidofovir for confirmed BKVN.

### 2.2. Statistical Analysis

The patients' demographic and laboratory values were compared between the stent and no-stent recipients. Results are expressed as proportion (%) or mean (standard deviation; SD). Analyses were performed with chi-square testing for categorical variables (Fisher's exact test used for violations of Cochran's assumptions) and Student's *t*-test for continuous variables (Mann-Whitney test was used for nonnormally distributed variables), respectively. Variables assessed in the stent and nonstent groups included recipient age group (<18, 18–50, or >50 years), recipient gender and race, donor source (living or deceased), cold ischemia time if deceased donor (<12, 12–24, or >24 hours), graft function (immediate or delayed), and induction agent (basiliximab versus Thymoglobulin). Statistical significance for univariate comparisons was defined as *P* < 0.05. Multivariate stepwise logistic regression models were fitted to study the adjusted relationship between UrSt use and the development of the outcome variables BKVM or BKVU by 12 months after transplant, the models including several covariates with *P* values < 0.10 on univariate testing, and induction agent, given the association our group has previously demonstrated between Thymoglobulin induction and subsequent BKV nephropathy [17]. Deceased donor source, higher cold ischemia time, and delayed graft function were strongly associated with each other, so only the latter two variables, more direct indicators of tissue injury, were included in the fitted models. Given the unexpected associations seen in our study group between stent placement and graft function, we also included an interaction term between UrSt placement and graft function in our multivariate models. All statistical analyses were performed using SAS software version 9.3 (Cary, NC, USA).

## 3. Results

A total of 640 kidney transplants were performed at the University of Florida/Shands Hospital between July 1, 2007, and December 31, 2010. The placement of ureteral stents or not was confirmed in 621/640 (97%). Thus we excluded 19 transplants, leaving a final study group of 621. Of these, 295/621 (47.5%) received a ureteral stent at time of kidney transplant. A total of 3283 urine samples with 583 positive tests (17.7%) and 8332 plasma samples with 934 positive tests (11.2%) were obtained from all the visits between months 1 and 12 after transplant.

The demographic parameters were not significantly different in the two groups (stent or no stent) with respect to recipient age, gender, ethnicity, or type of induction therapy (Table 1). Stents were inserted more frequently among recipients with a living donor transplant and low cold ischemia times, or immediate graft function, contrary to expectations [18].

When we performed a separate analysis restricting the study population to adults only (*n* = 581), the demographics did not change with respect to patterns seen in gender, ethnicity, donor source, or cold ischemia times. The only pattern changes were that proportion of induction agent (Thymoglobulin versus basiliximab) met statistical significance for difference, 60/45% in stent group versus 40/55% in nonstent group, *P* = 0.01. Immediate graft function, higher in the stented group, became borderline significant (*P* = 0.05). Further analyses were thus performed on the entire study group.

In univariate analysis models, ureteral stent placement was significantly associated with higher rates of BKVM and BKVU within the first year after transplantation (Table 1). The median onset for BKVM was 111 days (range 3–361) (75% of cases were in the first 6 months after transplantation) while the median for BKVU after transplant was 128 days (range 5–362) (72.2% of cases were in the first 6 months). Patient age, gender, and ethnicity were not statistically significant factors in the development of BK infection. As expected based on our study demographics, BKVM and BKVU rates were higher in recipient with IGF or with lower cold ischemia time (21% versus 32%, *P* = 0.03, and 16% versus 22%, *P* = 0.04), respectively.

In the multivariate model for BKVM, adjusting for multiple covariates as shown in Table 2, only UrSt placement remained as a significant variable (*P* = 0.04). Similarly, we included BKVU in the multivariate adjusted model, and again only UrSt placement remained as a significant variable (*P* = 0.04, Table 3).

The cutoff values currently used by many centers as significant enough to warrant intervention have changed over time. We ran additional analyses and determined that viremia >10,000 copies/mL was seen in 465 samples and viruria >10 million copies/mL was seen in 267 of the samples (approximately half of the total positives in each). At these cutoffs, none of the covariates identified as being significant previously retained significance in the univariate or multivariate analyses. However, our original hypothesis was that ureteral stent placement would increase the risk for any viremia or viruria, which was confirmed.

## 4. Discussion

The placement of a ureteral stent at time of kidney transplant offers the advantages of reduced stenosis, obstruction, or leakage across the newly created anastomosis between donor ureter and recipient urinary bladder. Conversely, the disadvantages of ureteral stent placement include possible migration of the stent, increased risk of early urinary tract infections, and the need for a separate urological procedure to remove the stent. In adults, many studies including two randomized trials suggested benefit, while others did not [19–27]. A meta-analysis by Wilson et al. showed that overall the benefits of preventing major urological complications exceeded the negatives from complications [23]. However, these studies and meta-analysis were performed before the association of stent placement with BK virus nephropathy was known, perhaps altering the risk-benefit ratio [15]. More recent single center analyses also looked at risk-benefit ratio without considering the potential for BKV replication or disease development [28]. In children, Bergmeijer et al. showed in the early 1990s a significant reduction of urological complications in stented versus nonstented children [29]. Ten years later, French et al. found no significant difference in both groups with overall lower urological complications than in prior eras [30].

Studies in animal models have revealed biological reactions to stent insertion in the form of epithelial destruction with erosions, ulcerations of the transitional epithelium, and inflammatory reactive changes within 6 weeks of stenting the animals' ureter [31–34]. Thus, the mechanical trauma associated with stent placement may injure the uroepithelium, allowing for latent BK virus to enter replicative phases. Atencio et al., in a mouse polyoma model, found that an injury was required for adult uroepithelial cells to be permissive for polyoma viral replication [33]. Other investigators created various mathematical models of BK viral infection dynamics and found that the model in which replication starts at the kidney and reaches the urinary tract, followed by bidirectional viral flux into both compartments, was the most compatible with clinical cases [35]. Hence, conditions that favor backwash vesicoureteral reflux, such as stenting, should be important in the dynamics of BK infection pathogenesis. Thus, clear pathophysiological mechanisms exist that make the association between stent placement and BKV infection plausible.

In this large, single center study, we observed* BKVM in 19% and BKVU in* 26% of the transplants, consistent with previous reports in the literature [13, 36]. Although the nonstented group had more patients receiving a graft from deceased donor and had higher ischemic times, the stent group still had a significantly higher adjusted risk for BKVM and BKVU. Multivariate analysis showed that ureteral stent placement was the only factor associated with an increase in the incidence of both BKVM and BKVU.

The major strength of this study is the large size of the cohort, the availability of detailed viral replication data both in the urine and blood, and a mix of pediatric and adults transplants. Notably, the prior studies associating stent placement with full BKVN comprised 200 [10] and 66 patients [11]. In conjunction with the prior associations with nephropathy which was noted in the two prior studies [10, 11], our results indicate that BKVN risk is therefore likely secondary to initiation of viral replication by the ureteral stent placement.

Prior studies associating stent placement with the precursor viremia stage comprised 186 [13] and 600 patients [14]. Our study, with 621 patients, looked at BK viruria, a precursor stage earlier to BK viremia, which was not done by both previous studies, though the second study was from our same institution, conducted independently by a separate author group [14]. Our study also found and adjusted for a significant association between immediate graft function and UrSt placement that the previous studies did not report and therefore they did not adjust for it in their multivariate analyses. Similar to the finding of other studies, we did not find any correlation between incidence of BKVM and the length of UrSt placement [13, 14]. The lack of correlation might indicate that the ureteral damage that occurs by UrSt placement is not related to stent duration. Furthermore, we did not find any correlation between induction antibody medication and BKVM. Thymoglobulin use was associated with BK in a UNOS database study [8], but dose details are not known in national databases and we use only a short 3-day induction, likely less than what others have used. Similarly, most of our recipients received tacrolimus, MMF, and prednisone, so it was not possible in our study to analyze if maintenance treatment has an effect on the risk of BKVM between both groups.

The limitations of our study include the single center and retrospective nonrandomized data collection. It is possible that the surgeon's decision to use a stent and the surgeon's competence may have depended on facing potential intraoperative ureteric complications that cannot be ascertained from chart review. In addition, regression analysis was used to account for five known confounding variables, namely, age, gender, ethnicity, induction type, and DGF, but other as yet unknown variables may have influenced this relationship. In our study, we found higher incidence of BKV infection in recipients with IGF. The reason for this association is not clear but we adjusted for it in our multivariate models. A randomized controlled trial in a large prospective study group is recommended to confirm our observations. Neither we nor others have attempted to determine if BKV PCR monitoring frequency or duration is altered by a finding of BK PCR positivity, the latter being more common after stent placement.

Our BKV screening protocol virtually eliminated early graft loss due to BK infection in our program without the use of an invasive allograft biopsy. In most of our BKV infection cases, PCR screening for BKV in the urine and/or blood allowed for early detection of the infection and with early intervention, progression of BKV infection to BKN was prevented. Ideally, these data are hypothesis-generating and randomized studies of ureteral stenting or not at time of kidney transplant are still needed to confirm such findings but may become unrealistic in light of the low allograft loss rates as many programs follow the guidelines for BK PCR monitoring.

In summary, our study demonstrates, in a large adult and pediatric population, that ureteral stent placement increases the risk for early precursor viral replication stages of BK virus allograft nephropathy in kidney transplantation. While the natural history and progression to full nephropathy is potentially modified by the use of frequent viral PCR monitoring and immunosuppression adjustment, the increased BKVM and BKVU incidence after ureteral stent placement may have changed the risk-benefit ratio that so far points to advantages of stent placement. This risk-benefit ratio needs to be revaluated.

## Figures and Tables

**Table 1:** Study group demographics based on stent placement.

Demographic variables	Stent placed	No stent	*P* value
Total number 621	295 (47.5%)	326 (52.5%)	
Age group			0.06
<18 yr	17 (6%)	15 (5%)
18–50 yr	139 (47%)	124 (39%)
>51 yr	139 (47%)	187 (75%)
Donor source			0.01
Deceased donor	231 (80%)	300 (92%)
Living donor	64 (20%)	26 (8%)
Gender			0.87
Male	190 (64%)	208 (64%)
Female	105 (36%)	118 (36%)
Race			0.59
White	171 (58%)	199 (61%)
African-American	99 (33%)	97 (30%)
Others	25 (8%)	30 (9%)
Induction			0.23
Thymoglobulin	81 (27%)	58 (18%)
Basiliximab	212 (72%)	262 (80%)
Missing	2 (0.7%)	6 (1.8%)
Cold ischemia time			0.02
<12 h	91 (31%)	58 (18%)
13–24 h	103 (35%)	113 (35%)
>24 h	99 (33%)	149 (45%)
Missing	2 (0.7)	6 (1.8%)
Graft function			0.02
Immediate	180 (61%)	166 (51%)
Delayed	112 (38%)	155 (47.5%)
Missing	3 (1%)	5 (1.5%)
BK viruria	75/250 (30.0%)	62/279 (22.2%)	0.04
137/529 (25.8%)			
BK viremia	63/289 (21.8%)	52/317 (16.4%)	0.05
115/606 (19.0%)			

**Table 2:** Logistic model for viremia.

Predictor variable	*P* value	Odds ratio (95% CI)
Ureteral stent	0.04	1.55 (1.04–2.38)
Age group	0.16	0.46 (0.14–1.55)
Gender	0.43	0.87 (0.55–1.36)
Cold ischemia time	0.42	1.62 (0.89–2.86)
Induction medication	0.82	0.82 (0.47–1.44)
Graft function	0.76	0.54 (0.31–0.98)

**Table 3:** Logistic model for viruria.

Predictor variable	*P* value	Odds ratio (95% CI)
Ureteral stent	0.04	1.67 (1.03–2.81)
Age group	0.13	1.39 (0.95–2.14)
Gender	0.49	1.15 (0.76–1.76)
Cold ischemia time	0.82	0.94 (0.55–1.61)
Induction medication	0.91	1.12 (0.66–1.92)
Graft function	0.41	0.65 (0.32–1.34)

## Abbreviations

BKV: BK virus
BKVN: BK virus nephropathy
BKVM: BK viremia
BKVU: BK viruria
DGF: Delayed graft function
IGF: Immediate graft function
MMF: Mycophenolate mofetil
PCR: Polymerase chain reaction
RTx: Renal transplant
UrSt: Ureteral stent.
